# Knee morphometric risk factors for acute anterior cruciate ligament injury in skeletally immature patients

**DOI:** 10.1007/s11832-015-0652-1

**Published:** 2015-03-28

**Authors:** K. Aaron Shaw, Brian Dunoski, Neil Mardis, Donna Pacicca

**Affiliations:** 1 Department of Orthopaedic Surgery, Dwight D. Eisenhower Army Medical Center, 300 East Hospital Road, Fort Gordon, GA 30905 USA; 2 Department of Radiology, Children’s Mercy Hospital, 2401 Gillham Road, Kansas City, MO 64108 USA; 3 Division of Orthopaedic Surgery, Children’s Mercy Hospital, 2401 Gillham Road, Kansas City, MO 64108 USA

**Keywords:** Anterior cruciate ligament, Pediatric sports medicine, Imaging—magnetic resonance, Anatomy

## Abstract

**Study design:**

Retrospective, case–control.

**Purpose:**

Knee morphometric risk factors for noncontact anterior cruciate ligament (ACL) injury have been a popular topic with skeletally mature patients. Little research has focused on the skeletally immature, with conflicting conclusions. This study performs a comprehensive analysis of identified parameters thought to predispose to ACL injury in a skeletally immature cohort.

**Methods:**

A retrospective review of pediatric patients undergoing knee magnetic resonance imaging (MRI) was performed over a 4-year period. Inclusionary criteria included mid-substance ACL disruption, skeletal immaturity, noncontact injury, without associated ligamentous disruption, and no medical condition associated with ligamentous laxity. MRI studies were analyzed by a pediatric musculoskeletal radiologist, measuring identified bony parameters, and compared with an age-matched control group without ligamentous injury. Data were analyzed using unpaired *t*-tests and logistic regression.

**Results:**

One hundred and twenty-eight patients sustained an ACL disruption, 39 met all inclusionary criteria (66 excluded for associated ligamentous disruption, 23 skeletally mature, three traumatic mechanisms, one with Marfan syndrome). When compared to an age-matched control cohort, the notch width index (NWI) was found to be significantly smaller in the ACL-injured group (*p* = 0.046). Subgroups analysis demonstrated significant differences in morphometric parameters between subjects with isolated ACL injuries and concomitant medial collateral ligament (MCL) strain.

**Conclusions:**

The NWI was significantly smaller in the ACL injury group. Significant differences were noted between isolated ACL injuries and ACL injuries with an MCL strain. This study further highlights the need for incorporating associated injury patterns when investigating the influence of morphometric factors for ACL injury in the skeletally immature.

**Level of evidence:**

Level III.

## Introduction

Anatomic risk factors for anterior cruciate ligament (ACL) disruption have been a recurring investigation in the orthopedic literature. Palmer [1] was the first to postulate an anatomic risk factor for ACL injury, suggesting stenosis of the intercondylar notch. Since this initial inquiry, numerous additional anatomical parameters have been investigated, finding an association with ACL injury and increased anterior–posterior knee laxity [2, 3], increased posterior sloping of the tibial plateau [4–6], shallow tibial plateau [5], decreased femoral condyle width [7, 8], an increase in the intercondylar notch volume [9], and a decrease in the height and volume of the tibial eminence [10].

With respect to the skeletally immature, few studies have investigated the influence of anatomic parameters on the predisposition to ACL injury. Domzalski et al. [11] investigated the impact of the intercondylar notch width index (NWI), finding that a decreased NWI was associated with ACL injury. They hypothesized that, as has been stated by numerous preceding authors, a smaller intercondylar notch width will exert greater forces at the mid-substance of the ACL, predisposing to injury. Vyas et al. [6] found a positive correlation for increased posterior slope of the medial tibial plateau and ACL injury in their skeletally immature cohort, but no significant effect for NWI.

To date, there has been no comprehensive study on a skeletally immature patient population with regard to the numerous anatomical parameters that have been previously identified. The purpose of this study was to perform a comprehensive review of identified morphometric parameters in a skeletally immature population, hypothesizing that decreased NWI and increased medial tibial plateau slope would be seen in the ACL-injured group when compared to an age-matched control cohort.

## Methods

Upon receiving approval from the Institutional Review Board, a review of all radiology reports for magnetic resonance images obtained of the knee performed at a pediatric specialty hospital between 1 January, 2009 and 1 January, 2013 was performed. Pediatric patients with an ACL injury were identified by ICD-9 code 844.2, in addition to CPT code 29888, identifying patients diagnosed with an ACL injury and those who had undergone an ACL reconstruction, and were cross-referenced against the magnetic resonance imaging (MRI) review. Reports that indicated the presence of an acute injury to the ACL were identified for an epidemiologic review of all injuries. Information including month of injury, age, gender, laterality of injury, and concomitant injury was collected.

### Study group

Identification of study participants was performed, selecting all subjects who were skeletally immature, defined as having open distal femoral and proximal tibial physes, without a complete concomitant ligamentous injury. A chart review of the identified subjects was then performed. Children with a traumatic mechanism of injury or history of a connective tissue disease associated with ligamentous laxity were excluded from participation. Additionally, incomplete lateral collateral ligament injuries were excluded due to association with posterolateral corner complex. Demographic information, including height, weight, and mechanism of injury, were collected.

**Fig. 1:**
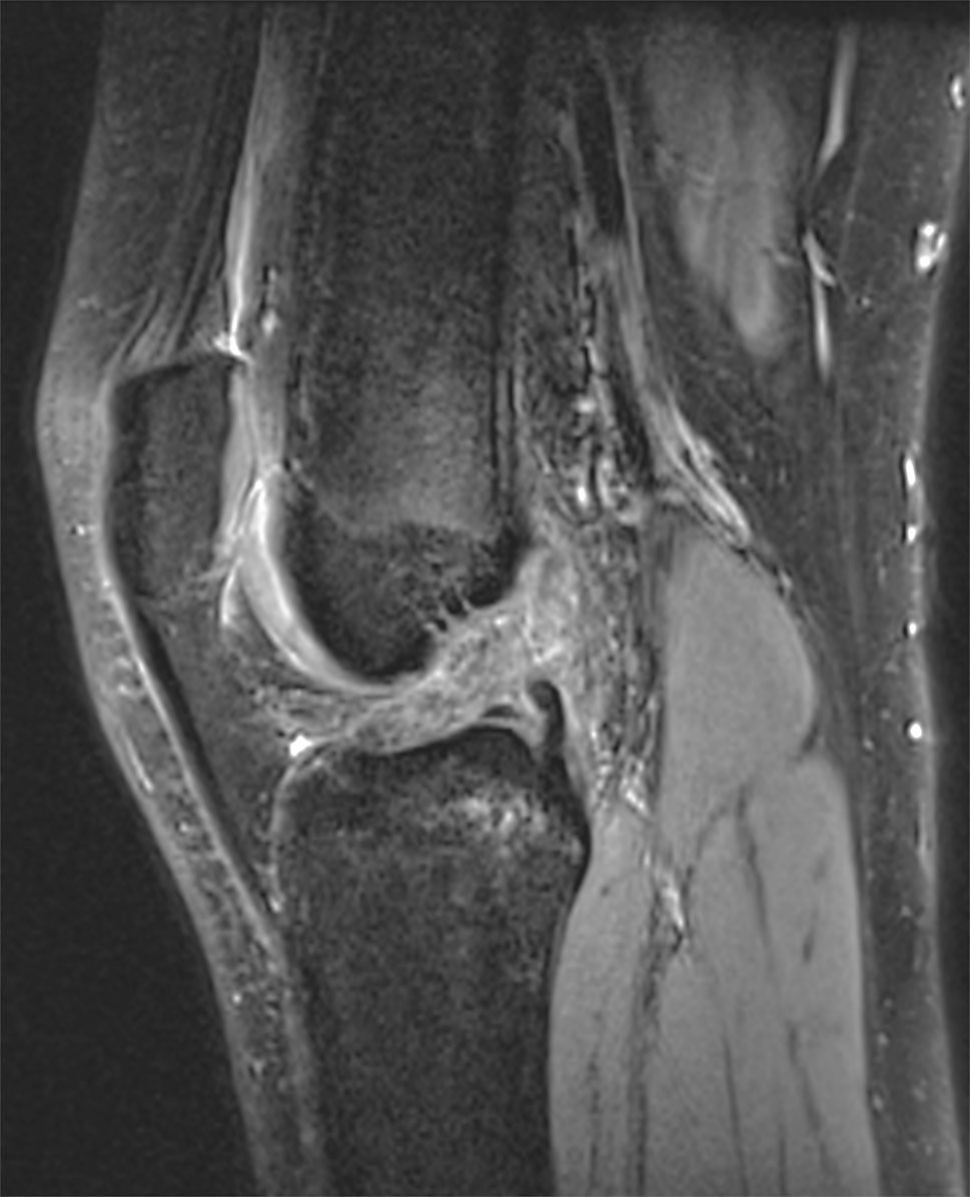
Sagittal image from fat-saturated, proton density sequence demonstrating disruption of the anterior cruciate ligament (ACL)

Following identification, the MRI sequences were analyzed by a pediatric musculoskeletal radiologist. Upon confirming the presence of disruption to the ACL (Fig. 1), measurements were performed of the tibia and the femur utilizing the annotation tools of the InteleViewer picture archiving and communication system (PACS) (Intelerad Medical Systems, Montreal, Canada).

**Fig. 2:**
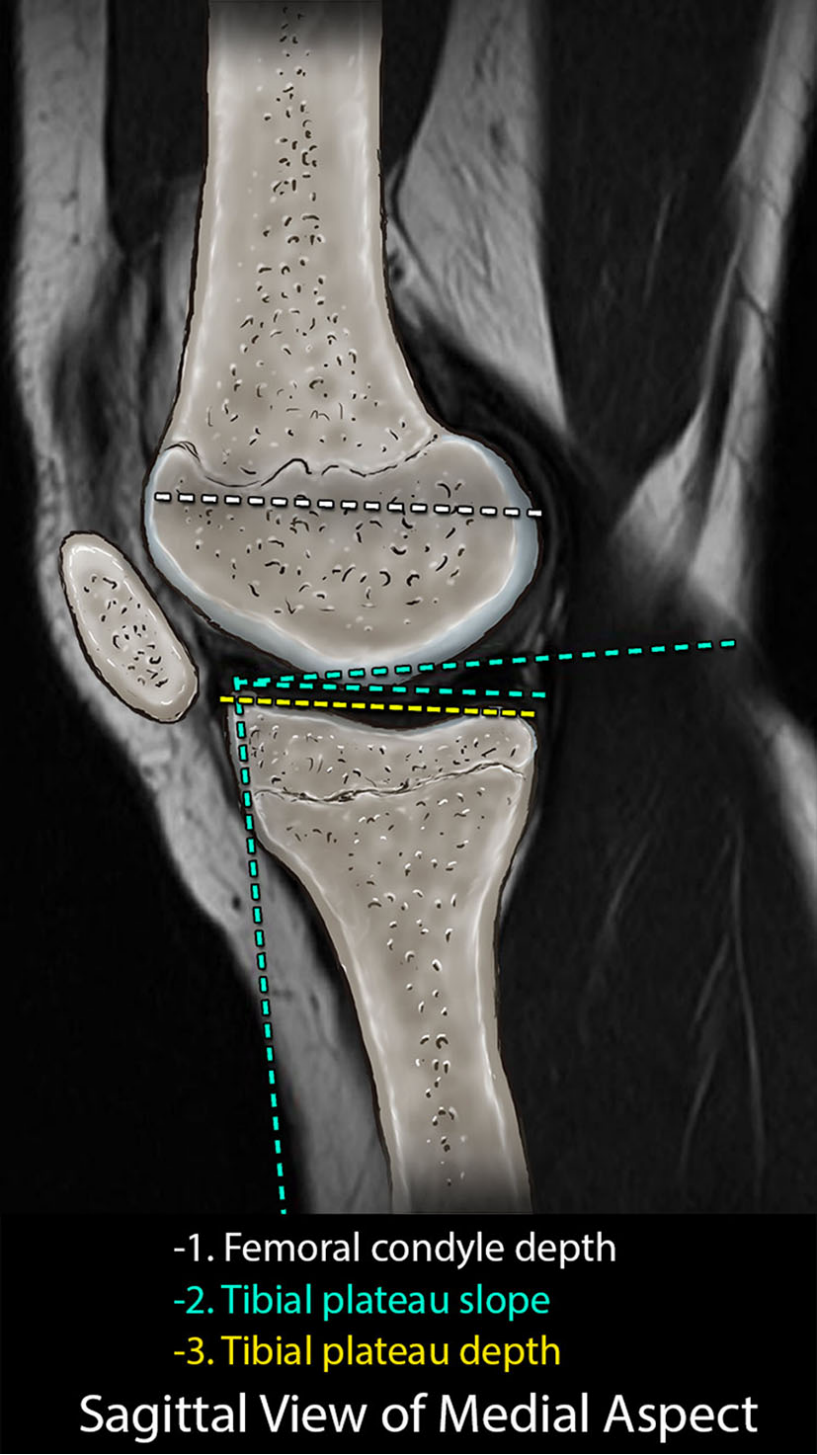
Representative sagittal image demonstrating the measuring techniques for the medial and lateral tibial plateau slope and depth

**Fig. 3:**
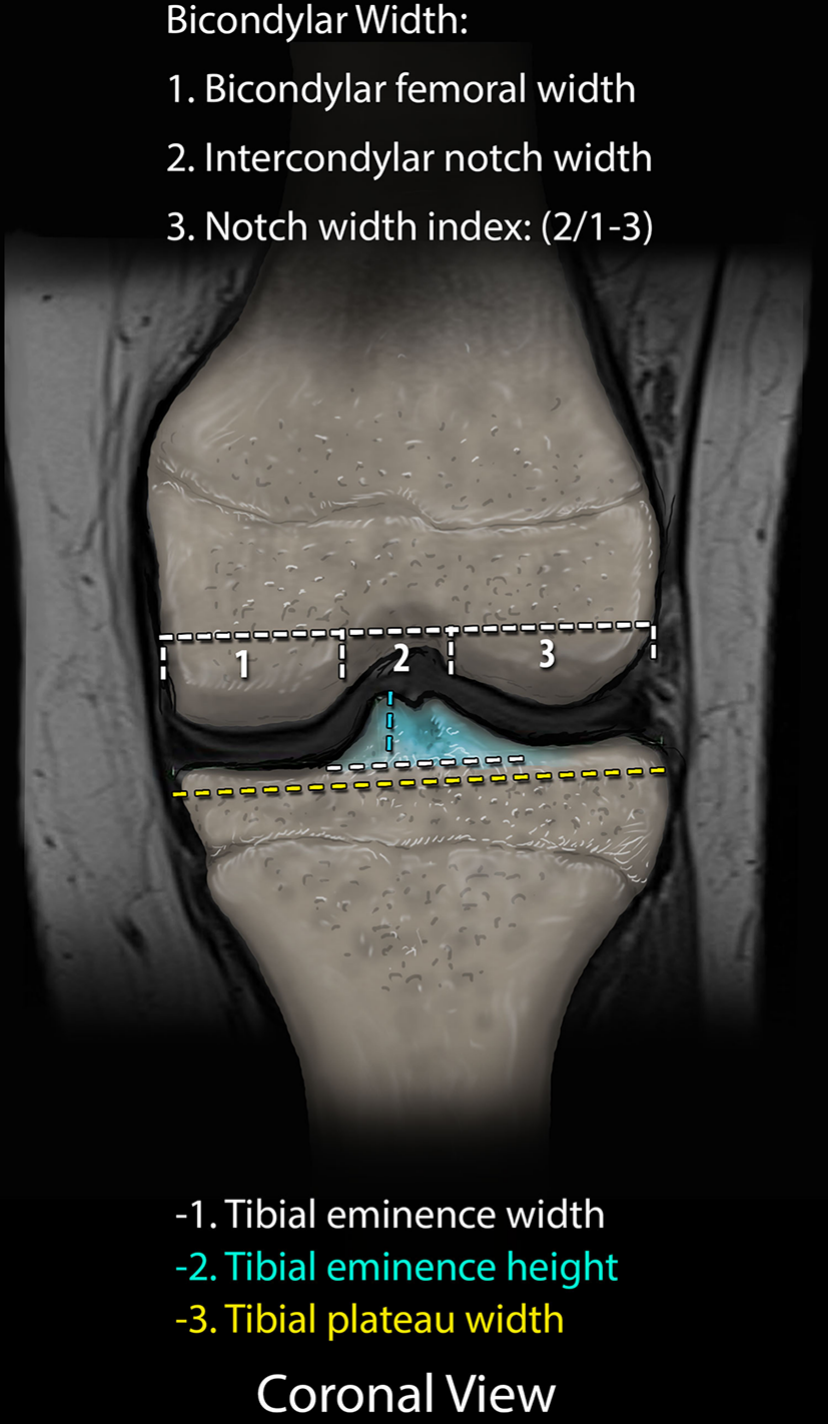
Representative coronal image demonstrating the measurement techniques for the intercondylar notch width, notch width index (NWI), and tibial eminence height and width

**Fig. 4:**
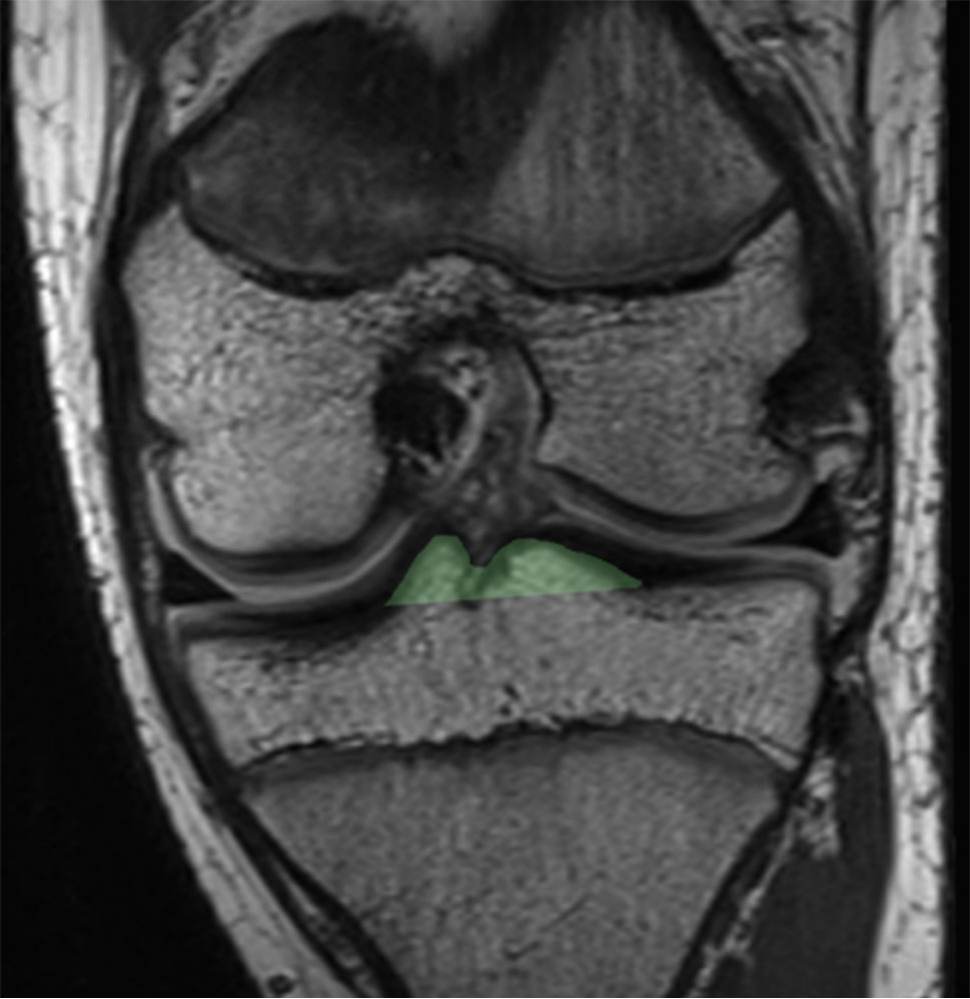
A single coronal image of a proton density (PD) magnetic resonance imaging (MRI) sequence demonstrating the measuring technique for the tibial eminence volume. The measuring technique was applied to all coronal slices of the tibial eminences and summated to generate the volumetric data

Utilizing sagittal plane images, the posterior slope and depth of the medial tibial plateau were assessed as described by Hashemi et al. [10] (Fig. 2). Coronal plane images were then analyzed to determine the widest medial–lateral width of the tibial plateau, which was recorded as the tibial plateau width. Assessment of the width, height, and volume of the tibial eminences was determined using the technique of Hashemi et al. [10] (Fig. 3). Volumetric analysis of the tibial eminences was performed, using the InteleViewer volumetric annotation tool, outlining the bony content of the tibial eminence, defined by the tibial plateau reference line and consisting of the bony architecture intersecting the reference line (Fig. 4). This technique was reproduced for each image containing the tibial eminences and a volumetric calculation was generated.

**Fig. 5:**
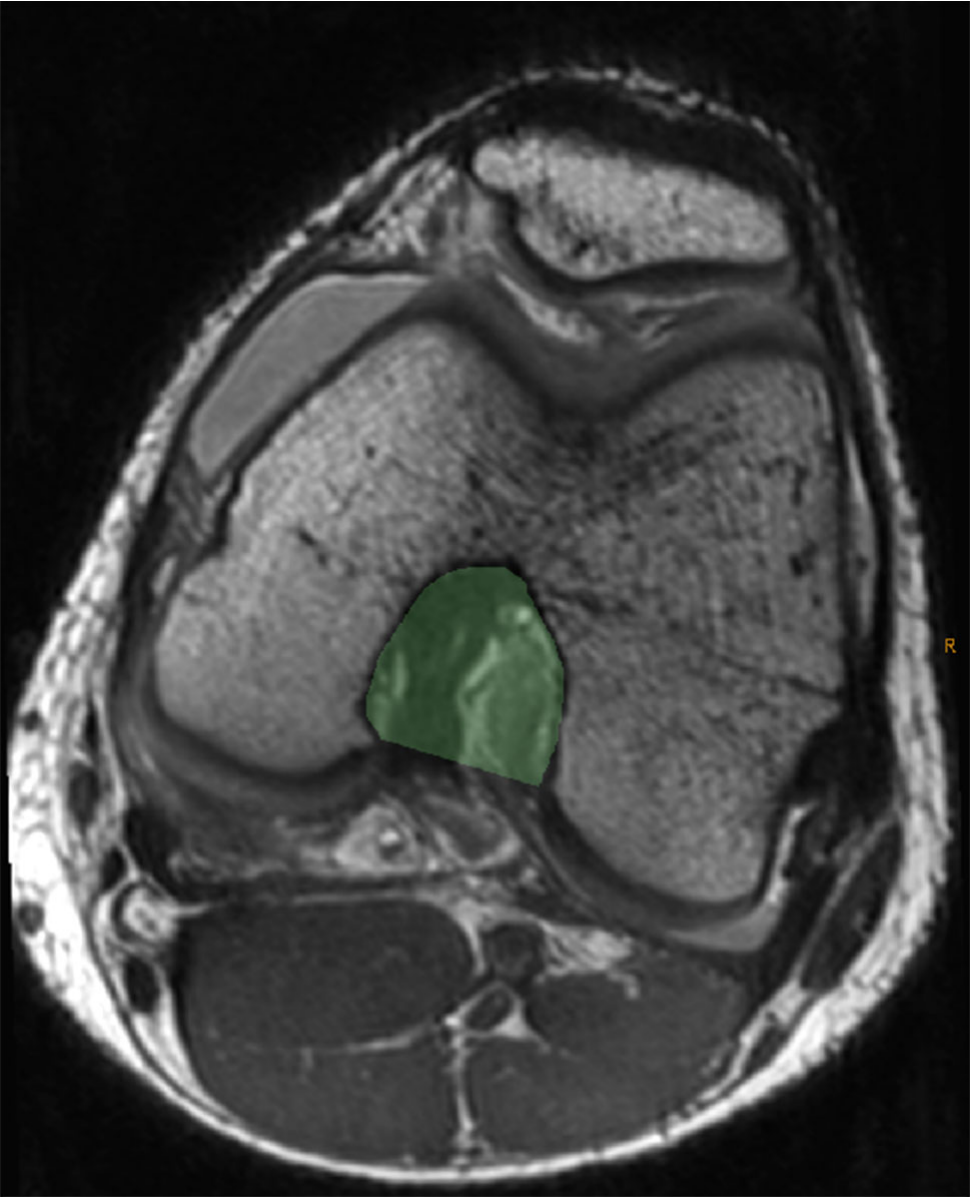
Axial image of a PD MRI sequence demonstrating the measuring technique for the intercondylar notch volume. The measuring technique was applied to all axial images included within the defined borders of the femoral notch and summated to generate the volumetric data

Femoral measurements included the intercondylar notch width, bicondylar width, NWI, medial and lateral femoral condyle width, and intercondylar notch volume. The NWI was determined as described and validated by Vrooijink et al. [8] with measurement of the femoral intercondylar notch width, standardized to the overall bicondylar femur width (Fig. 3). The axial images were used to calculate the notch volume using the technique of Charlton et al. [13], using the InteleViewer volumetric annotation tool (Fig. 5).

### Control group

Following identification of study participants, a gender- and age-matched control cohort of children with a statistically similar median age (*p* < 0.05) to the study group, in whom a knee MRI was performed as part of their routine care, were identified. Children who were skeletally immature with an intact ACL and no associated ligamentous injury met the inclusionary criteria. MRI sequences where then analyzed and measurements were performed of the defined tibial and femoral morphometric parameters using the aforementioned protocol. Additionally, measurements were performed to assess the width and cross-sectional area of the ACL at its mid-substance.

### Statistical analyses

Statistical analyses were performed using SAS statistical software version 9.2 (SAS Institute Inc., Cary, NC, USA). Data from the experimental group were analyzed in whole, as well as in subgroups consisting of isolated ACL injury and ACL injury with medial collateral ligament (MCL) strain (ACL/MCL). Unpaired *t*-tests, comparing the injured subject population to the controls, were performed for each measurement. Logistic regression was performed, using a model consisting of the medial tibial plateau slope, medial tibial plateau depth, NWI, intercondylar notch volume, tibial eminence volume, and tibial eminence height and width to determine if these variables were predictive of ACL injury. These analyses were repeated for each subgroup. Using gender as an independent variable, the GLM procedure was utilized to perform analyses of variance for each parameter to identify potential differences between the isolated ACL injury and control groups. Statistical significance was predetermined as *p* < 0.05. Additionally, the control patient data were utilized to perform cross-assessments of the notch width, NWI, ACL width, ACL area, tibial eminence width, and tibial eminence volume using Pearson correlation coefficients.

## Results

A total of 128 (74 male, 54 female, average age 15.27 years) acute ACL disruptions were identified during the selected 4-year timeline. Of the 128 ACL disruptions, 62 (48.4 %) affected the right knee, 71 (55.5 %) affected the left, and a total of 31 (24.2 %) occurred in isolation. Of the 128 total ACL disruptions, only 66 exhibited no associated ligamentous injury and were considered for inclusion in the study. Following imaging review, 23 had attained skeletal maturity as determined by closed distal femoral and proximal tibia physes on MRI. Four additional children were excluded during chart review, three secondary to a traumatic mechanism of injury reported during their initial orthopedic clinic visit and one additional child with a diagnosis of Marfan syndrome, leaving a total of 39 children for study participation, 28 without associated ligamentous injury and 11 with associated MCL strain.

The average age of the injured study group was 14.245 years (±2.08), consisting of 18 females and 21 males. The control group was composed of 28 children (14 females, 14 males), with an average age of 14.29 years (±1.08). The results of the between-group *t*-tests for the various radiographic parameters are summarized in Table 1 for all identified patients. The NWI and medial tibial plateau depth were found to be significantly different from the control group, with the injured group demonstrating decreased NWI and increased medial tibial plateau depth. Logistic regression analysis found the NWI to be marginally predictive (*p* = 0.052) of ACL injury. Post-hoc power analysis demonstrated an effect size of 1.34, with an achieved power of 0.96.

**Table 1:** Results of unpaired *t*-tests of radiographic parameters for identified patients with an anterior cruciate ligament (ACL) injury compared to age-matched control patients, with results of subgroup comparisons and gender analysis of assessed parameters

Parameter	ACL vs. control	Isolated ACL vs. control	ACL/MCL vs. control	Isolated ACL vs. ACL/MCL	Gender analysis
Notch width	0.19	0.22	0.21	0.004*	0.2
Notch width index	0.046*	0.009*	0.79	0.026*	0.22
Notch volume	0.73	0.41	0.02*	0.002*	0.01*
MTP slope	0.50	0.95	0.04*	0.18	0.19
LTP slope	0.45	0.62	0.39	0.55	0.19
MTP depth	0.01*	0.063	0.01*	0.14	0.08
LTP depth	0.26	0.55	0.03*	0.20	0.31
TE height	0.83	0.60	0.09	0.07	0.005*
TE width	0.48	0.18	0.50	0.054	0.01*
TE volume	0.77	0.53	0.04*	0.023*	0.0003*

*MTP* medial tibial plateau, *LTP* lateral tibial plateau, *TE* tibial eminence

* Denotes statistical significance at *p* < 0.05

For isolated ACL injuries, the NWI was the only parameter found to reach statistical significance (*p* = 0.009), with the isolated ACL-injured subjects demonstrating decreased NWI compared to the controls. On logistic regression analysis, the NWI was not predictive of ACL injury (*p* = 0.066). Analysis of the ACL/MCL subgroup demonstrated significant differences, with a larger intercondylar notch volume and smaller tibial eminence volume, medial tibial plateau slope and depth, and lateral tibial plateau depth when compared to the control group. Due to the small sample size, logistic regression analysis was not performed. Subgroup comparison revealed the isolated ACL injury group to have significantly smaller intercondylar notch widths, NWI, intercondylar notch volume, and tibial eminence volume.

Pearson correlations were performed on control subjects to determine the degree and type of relationship between the intercondylar notch width, NWI, intercondylar notch volume, ACL width, ACL area, tibial eminence width, and tibial eminence volume. As seen in Table 2, significant positive correlations were found between the notch width and all other variables (*p* < 0.05). The intercondylar notch volume was statistically significant with all other measures, except for the ACL width and ACL mid-substance cross-sectional area; however, it did approach statistical significance with the ACL area (*p* = 0.052). The NWI had a significant positive correlation with the ACL width (*p* < 0.05), but not with the ACL area or the tibial eminence width or volume. As expected, there were strong positive correlations between the ACL width and ACL area, and between the tibial eminence width and area (all *p* < 0.05). However, no correlations were found between the ACL measures and the tibial eminence measures (all *p* > 0.05).

**Table 2:** Pearson correlation data performed on the control patients

	NW	NWI	Notch volume	ACL width	ACL area	TE width	TE volume
NW		0.801**	0.587*	0.419*	0.422*	0.407**	0.575**
NWI			0.220	0.397**	0.259	0.152	0.218
Notch volume				0.254	0.371^a^	0.383**	0.630**
ACL width					0.796**	0.76	0.217
ACL area						0.240	0.294
TE width							0.549**

*NW* notch width, *NWI* notch width index, *ACL* anterior cruciate ligament, *TE* tibial eminence

* Significant at 0.05

** Significant at 0.005

^a^Marginally significant at 0.052

The results of the *t*- and *F*-test analyses (Table 1) indicated significant differences based on gender, with female patients demonstrating decreased intercondylar notch volume (*p* = 0.01, *F* = 7.11), tibial eminence height (*p* = 0.0054, *F* = 8.37), tibial eminence width (*p* = 0.0141, *F* = 6.43), and tibial eminence volume (*p* = 0.0003, *F* = 14.66). There were no significant gender differences in the ACL width or ACL cross-sectional area as assessed in the control population.

## Discussion

Since Palmer’s first postulation of intercondylar notch stenosis predisposing to ACL injury [1], numerous potential anatomic risk factors have been suggested, to include increased posterior sloping of the tibial plateau [4–6], shallow tibial plateau [5], decreased femoral condyle width [7, 8], an increase in the intercondylar notch volume [9], and a decrease in the height and volume of the tibial eminence [10]. The majority of investigations have isolated their investigations to adult and young adult patient populations. Domzalski et al. [11] found a correlation between decreased NWI and a predisposition to ACL disruption in a skeletally immature population, determined by open physes on MRI. Vyas et al. [6] found no correlation with the NWI, but did find a positive correlation between the posterior tibial slope and ACL injury. To date, there has been no comprehensive review of identified anatomic risk factors or an analysis subdivided by injury pattern.

Our investigation identified ACL-injured patients to demonstrate significantly smaller NWI when compared to the control group. However, when carried out through logistic regression, this was not predictive of ACL injury, although it did approach statistical significance (*p* = 0.052). When this analysis was carried out for the subgroup analyses, the NWI remained significantly smaller in the isolated ACL-injured group; however, the ACL/MCL group demonstrated no difference in the NWI. Larger notch volumes and smaller medial tibial slopes and tibial plateau depths were present in this subgroup when compared to the control group. None of these parameters were found to be predictive of injury by logistic regression analysis. When compared, the isolated ACL subgroup had significantly smaller intercondylar notch width, NWI, notch volume, and tibial eminence width than the ACL/MCL subgroup.

These findings may, in part, help to elucidate the tremendous variability reported in the literature regarding bony parameters and the predisposition for ACL injury. Neither Vyas et al. [6] or Domzalski et al. [11] included information regarding associated injuries in their identified cohorts. Our findings demonstrate significant differences in bony parameters between identified subgroups, with the ACL/MCL subgroup demonstrating no difference in the NWI when compared to the control group, whereas the isolated ACL subgroup had significantly smaller NWI. As such, if previous investigations contained a disproportionate representation of one subgroup over another, the statistical analyses would represent this accordingly.

The majority of research investigating the influence of intercondylar notch width is founded upon the presumed correlation of the notch width and the size of the ACL [14]. Muneta et al. [15] found no correlation between the intercondylar notch width and ACL size in a cadaveric study of 16 skeletally mature knees. However, Stijak et al. [16] revisited this investigation using a larger sample of 50 skeletally mature cadaveric knees, finding a significant correlation in males between the intercondylar notch width and ACL width. In our investigation with a skeletally immature population, we found a significant correlation between the intercondylar notch width and NWI, ACL width, and ACL area, as well as the NWI and ACL width using measurements of the control group. These findings suggest that, in the skeletally immature patient, the width of the intercondylar notch does correlate with the width and cross-sectional area of the ACL, supporting the hypothesis that notch stenosis may predispose to ACL injury.

Gender is one factor that is believed to be involved in the incidence of ACL injury. Previous epidemiologic studies have identified a six-fold greater risk of ACL injury in female collegiate soccer players compared to their male counterparts [17]. Hormonal risk factors are believed to serve an important role [18]; however, there is an insufficient body of evidence to obtain a strong consensus [19]. We identified gender differences in the bony morphology, which may be an additional factor in the predisposition to injury. Female subjects demonstrated decreased intercondylar notch volumes, as well as tibial eminence height, width, and volume, when compared to their male counterparts.

The impact of skeletally immaturity on the ACL is not fully understood. An intrasubstance disruption of the ACL had previously been thought to be a rare occurrence in the skeletally immature [20]. Whereas tibial spine avulsion fractures were thought to be more characteristic of ACL injury in the skeletally immature, increasing competitive sport participation [20, 21], single-sport concentration [20], as well as differences in knee loading and relative strength of the tibial eminence have all been implicated as contributors to this paradigm shift. Previous studies have demonstrated alterations in articular cartilage in response to athletic training [22, 23]. A recent animal study on thoroughbred horses demonstrated increased bone density in the epiphysis of the 3rd metacarpal in response to training [24]. Increased sport training could result in strengthening of the physeal cartilage or increased bone formation to the tibial eminence, with the single-sport focus producing an imbalance in the neuromuscular control of the knee, predisposing to a mid-substance ligamentous injury. As to the topic of bony morphology, no longitudinal studies to date have investigated changes, if any exist, to bony and ligamentous parameters during maturation.

There are several limitations to our study. The retrospective design has some inherent biases. The data obtained from the chart review are limited to the thorough nature of the initial input. Body mass index (BMI), height, and weight were designed to be included in the analysis. However, due to the lack of sufficient datasets in the control subjects at the time the MRI was obtained, this analysis was abandoned. Additionally, the control group was limited by the number of patients requiring MRI evaluation of their knee for reasons unrelated to a ligamentous injury. The possibility of selection bias could be present, as all patients were treated at a pediatric specialty hospital. Imaging studies included in the study were isolated to studies performed at our institution to minimize measurement error in comparing studies produced by magnets of varying strengths or using different sequencing protocols. Many patients who are referred to our institution for an ACL disruption present after already having obtained a magnetic resonance image. Patients with established care may be more likely to have had their studies performed at our institution and, thus, be included in the study. Additionally, the possibility of measurement bias cannot be excluded. The measurements were performed by a single radiologist and blinding to the patient group was not able to be incorporated given the nature of the study design. Finally, a lack of power could be influencing the lack of statistical significance for defined parameters.

## Conclusion

In summary, anterior cruciate ligament (ACL) disruptions are an increasingly common occurrence in the skeletally immature patient. This is the first study to perform subgroup analyses of previously identified morphometric parameters thought to predispose to ACL injury in a skeletally immature population. We identified a statistically significant difference in the notch width index (NWI) between the injury and control groups that was marginally predictive of ACL injury. Additionally, we identified differences in the identified parameters between isolated ACL injuries and ACL injuries with concomitant medial collateral ligament (MCL) strain. This information identifies the need for subgroup analyses when assessing the influence of bony parameters on the predisposition to ACL injuries in the skeletally immature.
